# RE: Should We Use Rifampicin in Periprosthetic Joint Infections Caused by Staphylococci When the Implant Has Been Exchanged? A Multicenter Observational Cohort Study by Kramer et al.

**DOI:** 10.1093/ofid/ofae074

**Published:** 2024-02-08

**Authors:** Heime Rieber

**Affiliations:** Division of Microbiology, MVZ Dr. Stein and Colleagues, Mönchengladbach, Germany

To  The  Editor—I read with interest the article by Kramer et al. published in *Open Forum Infectious Diseases* [1]. In a retrospective multicenter observational study, the authors investigated the use of rifampicin in periprosthetic joint infections (PJIs) caused by *Staphylococcus* species after implant exchange. A subanalysis of chronic PJIs that were treated by 2-stage exchange arthroplasty demonstrated a lower failure rate during follow-up if rifampicin had been administered, compared with a nonrifampicin group. According to the authors, the benefit was predominantly observed in chronic infections caused by *S. aureus*. I have some comments on this.

*Staphylococcus aureus* causes a wide range of human diseases. In connection with joints and joint prostheses, this microorganism is mainly associated with acute infections. A relapse or chronic infection with *S. aureus* generally leads to the formation of subpopulations, designated small-colony variants (SCVs), which may persist intracellularly in the host tissue for a long time due to reduced expression of virulence factors.

Administering antibiotics that do not penetrate sufficiently into the host cells or metabolic mechanisms of SCVs may lead to the emergence of resistance, for example, against aminoglycosides and trimethoprim-sulfamethoxazole [2].

The extent to which particular SCVs were identified both in the rifampicin group (n = 37) and in the nonrifampicin group (n = 31) is not apparent from the study data. But it would be important to know because chronic infections with SCVs are always special individual cases with a very specific pathogen–host interaction that cannot simply be summarized in a group [3].

Furthermore, detection of SCVs in laboratory tests must be communicated to the clinician. Due to slow growth in vitro and depending on the measurement methods used, the laboratory is often unable to reliably determine the minimal inhibitory concentration (MIC) of numerous antibiotics. This in turn means that the measured values may be reported as incorrectly sensitive. Therefore, MICs should be evaluated with caution and discussed with the microbiologist because they may have an impact on antimicrobial therapy.

A different constellation is involved if the chronic infection occurs as a re-infection with another *S. aureus* strain, for example, related to a postoperative wound infection. In this case, a causal relationship with the initial infection cannot be established with absolute certainty, so the effectiveness of the primary antimicrobial therapy cannot be determined.

Finally, we do not know which antibiotics in which dosage, single or in combination, were used in the nonrifampicin group and why rifampicin was not used in this particular group. But it would be desirable in order to better understand why the antimicrobial therapy was not successful. Ultimately, the central message of this study relates to the higher failure rate in this group (16/31), indicating that administration of rifampicin was a significant benefit for the patients in the rifampicin group, who experienced a lower failure rate (9/37).

The authors themselves indicated that they could not provide information on the duration, dosage, and combination partner of rifampicin. Therefore, whether the reported effect is significant cannot be conclusively determined.

In conclusion, this observational study contains too many ambiguities to clarify the therapeutic benefit of rifampicin against *S. aureus* in chronic PJI.
